# Developing and validating a Japanese version of the Weight Self-Stigma Questionnaire

**DOI:** 10.1007/s40519-023-01573-0

**Published:** 2023-05-17

**Authors:** Yuko Nakamura, Michiko Asano

**Affiliations:** 1 Center for Evolutionary Cognitive Sciences, Graduate School of Art and Sciences, The University of Tokyo, 3-8-1, Komaba, Meguro-Ku, Tokyo, 153-8902 Japan; 2 University of Tokyo Institute for Diversity & Adaptation of Human Mind (UTIDAHM), Meguro-Ku, Tokyo, 153-8902 Japan

**Keywords:** Weight bias internalization, Weight Self-Stigma Questionnaire, Weight stigma, Obesity

## Abstract

**Purpose:**

Weight bias internalization (WBI) is significantly associated with negative physiological and psychological consequences. Given its negative effects, appropriate measurement of WBI is required for weight management and mental and physical health in people with weight problems. One of the most reliable and frequently used questionnaires to assess WBI is the Weight Self-Stigma Questionnaire (WSSQ). However, a Japanese version of the WSSQ has not yet been developed. Thus, the current study aimed to develop a Japanese version of the WSSQ (WSSQ-J) and validate its psychometric properties in the Japanese context.

**Methods:**

A total of 1454 Japanese participants (age 34.44 ± 6.92; male = 498) with diverse weight statuses (Body mass index: 21.44 ± 3.52, 13.79–41.40 kg/m^2^) completed an online survey for the WSSQ-J. The internal consistency of the WSSQ-J was estimated by calculating Cronbach’s α. Confirmatory factor analysis (CFA) was then carried out to confirm that the factor structure of the WSSQ-J was the same as that of the subscales of the original WSSQ.

**Results:**

The WSSQ-J had a Cronbach’s α of 0.917, indicating good internal consistency. In CFA, the comparative fit index was 0.945, the root mean square error of approximation was 0.085, and the standardized root mean square residual was 0.040, indicating that the two-factor model showed satisfactory goodness-of-fit.

**Conclusion:**

This study replicated the original findings related to the WSSQ, showing that the WSSQ-J is a reliable WBI questionnaire consisting of two factors. Therefore, the WSSQ-J would be a reliable tool for assessing WBI among Japanese.

**Level of evidence:**

Level V, descriptive cross-sectional study.

**Supplementary Information:**

The online version contains supplementary material available at 10.1007/s40519-023-01573-0.

## Introduction

It is becoming increasingly clear that people with greater body mass index (BMI) are facing weight-related bias or stigma [1, 2]. Weight bias is defined as the inclination to form unreasonable judgments or negative ideologies based on a person’s weight [3], such as beliefs that individuals with greater BMI are lazy, sloppy, incompetent, and lack willpower [4, 5]. Weight bias can lead to weight stigma [6], which refers to discriminatory acts and attitudes targeted towards individuals because of their weight and size [7, 8]. Weight stigma can affect an individual’s access to education, employment, and medical care [9–11], causing health and social inequalities.

Weight bias internalization (WBI) is often a result of exposure to weight stigma [12], and occurs when individuals apply negative weight stereotypes to themselves and self-derogate because of their body weight [5]. WBI is observed in various countries [13, 14] and is higher among people who are female, younger, and have a higher BMI [15, 16]. WBI is related to depression, anxiety, body image, self-esteem [5, 17, 18], and disordered eating in adults [16, 19–22] with any body weight status, including underweight/average weight, overweight, obese [23], and also in children [24]. Further, WBI could elevate body dissatisfaction [23], which is connected with impaired psychological and biological health [25], and unhealthy eating and negative health behavior [26]. Such disordered eating would relate to increased risk of metabolic syndrome [17] and difficulty maintaining a moderate weight [13, 27]. In addition, WBI is connected to poor mental and physical health-related quality of life [13, 28, 29].

Given such negative effects of WBI on affected individuals, appropriate WBI measurement is required for weight management and improving mental and physical health in people suffering from WBI. Additionally, such a measure is needed for the evaluation of WBI reduction interventions. To assess WBI, two self-reported questionnaires, the Weight Bias Internalization Scale (WBIS) and the Weight Self-Stigma Questionnaire (WSSQ), were mostly used in previous studies [5]. The WBIS was designed to assess the internalization of weight bias among individuals who are overweight and obese; it measures the internalization of negative characteristics that these people attribute to themselves [30]. The WSSQ assesses weight self-stigma and was designed to capture the multi-dimensional nature of WBI, self-devaluation, and fear of enacted stigma in populations of individuals who are overweight and obese [31]. A previous systematic review identified approximately 40 original self-report weight stigma questionnaires and evaluated their psychometric properties [32]. Among these questionnaires, the WSSQ had the most extensive psychometric validation [32]. The WSSQ shows good psychometric properties and validity and has been widely used to assess WBI in previous research [5, 33]. In addition, although the original WSSQ was adapted for people who are overweight or obese [31], unlike the WBIS, the WSSQ has been shown to be measurement invariant in “overweight” and “non-overweight” pediatric (aged 8 to 12 years) [34] and young adult populations [35], and in young adults with a broad range of weight status from underweight to overweight [36].

The rate of eating disorders is increasing among the Japanese population [37, 38]. Japanese females are at an especially high risk of suffering from eating disorders [39, 40]. Eating disorders (e.g., anorexia nervosa, bulimia nervosa, and binge-eating disorder) are characterized by disordered eating (e.g., dieting for weight loss, binge eating, and self-induced vomiting) [41], and disordered eating can evolve into eating disorders [42]. Therefore, because WBI is related to disordered eating [16, 19–24], eating disorders could potentially be associated with WBI. Given that sociocultural pressures related to appearance are involved in WBI [43] and the prevalence of eating disorders are on the rise in Japan [37, 38], an appropriate WBI measurement in the Japanese context is required. However, a Japanese version of the WSSQ has not yet been developed. Therefore, the current study aimed to develop a Japanese version of the WSSQ (WSSQ-J) and validate its psychometric properties in the Japanese context.

## Methods

### Participants

Given that most adult patients with eating disorders in Japan are aged 20–45 years [37, 44, 45], participants aged 18–45 years were included in the online survey. 100nin Enquete, a commercial survey sampling and administration company, was contracted to recruit participants and implement the online-based survey, which was conducted on June 11, 2022–June 13, 2022. Samples were acquired from existing pools of general Japanese participants. To improve the data quality of the survey, we added two dummy questions in the questionnaire. They were questions asking respondents to select a specific answer to confirm whether the respondents actually read and understood the questions. Originally, 1509 participants completed the survey; 55 participants who failed to answer the dummy questions were excluded and a total of 1454 Japanese participants between 18 and 45 years [age 34.44 ± 6.92 (mean ± standard deviation); male = 498, female = 956] were included in the statistical analyses (Table 1). The current study was approved by the Ethics Committee of the Department of Arts and Sciences, University of Tokyo (Approval No. 812-2). At the beginning of the online survey, participants were asked for their consent to contribute their data for research purposes, and we included participants who consented to participate in this research.

**Table 1:** Demographic characteristics (n = 1454)

Variables	Mean ± standard deviation (range)
Age	34.44 ± 6.92 (18.0–45.0)
BMI^a^	21.44 ± 3.52 (13.79–41.40)
Non-overweight (n = 1256) (BMI < 25)	20.41 ± 2.20
Overweight (n = 157) (BMI = 25.0 to < 30)	26.80 ± 1.41
Obese (n = 39) (BMI = 30.0 or higher)	33.14 ± 3.10
Sex: Male:Female	498:956
WSSQ-J (total)	29.92 ± 10.14 (12–59)
WSSQ-J (self-devaluation)	15.78 ± 6.39 (6–30)
WSSQ-J (fear of enacted)	14.14 ± 4.59 (6–30)
TFEQ-J (cognitive restraint)	8.39 ± 4.16 (0–21)
TFEQ-J (disinhibition)	7.04 ± 3.79 (0–16)
TFEQ-J (hunger)	4.80 ± 3.11 (0–14)

The minimum and possible maximum scores for each questionnaire were as follows: WSSQ-J (total): 12–60, WSSQ-J (self-devaluation): 6–30, WSSQ-J (fear of enacted): 6–30, TFEQ-J (cognitive restraint): 0–21, TFEQ-J (disinhibition): 0–16, and TFEQ-J (hunger): 0–14

^a^BMI excluded two missing values

Participants completed the WSSQ-J, and the Japanese version of the Three-Factor Eating Questionnaire (TFEQ-J), and provided their age (years), sex, height (cm), and weight (kg). Self-reported height and weight were used to calculate the individual BMI.

### Measures

#### The Japanese version of the WSSQ

With permission from the author of the original WSSQ [31], the WSSQ-J was developed according to the following standard procedures [46]. First, the original WSSQ was translated into Japanese by two Japanese researchers with high English proficiency. Second, the translated WSSQ was back-translated by a professional English editing and translation company (Editage; https://www.editage.com). Third, the author of the original WSSQ [31] confirmed that the back-translated WSSQ was conceptually and linguistically equivalent to the original WSSQ.

The WSSQ-J is a 12-item measure of weight-related self-stigma [31] (Supplemental Table S1). An example item reads, “I feel guilty because of my weight problems.” Each item of the WSSQ is rated on a 5-point Likert scale, with scores ranging from 1 (completely disagree) to 5 (completely agree). Sum scores are calculated for the full scale and each subscale. Items 1–6 constitute the self-devaluation subscale, and items 7–12 constitute the fear of enacted stigma subscale. There are no reverse-scored items. A high WSSQ-J score denotes a high level of weight-related self-stigma.

#### The Japanese version of the TFEQ

In the original study of the WSSQ, each subscale of TFEQ showed significant positive correlations with the total and subscales of the WSSQ [31]. Therefore, we examined associations between TFEQ-J and WSSQ-J.

The TFEQ-J is a 51-item measure of eating behavior [47, 48]. The items consist of 36 closed questions with a forced, true/false response and 15 Likert rating items. The TFEQ-J measures three aspects of eating behavior: (i) “cognitive restraint” as the degree of cognitive control of daily food intake, (ii) “disinhibition” as loss of control of food intake, and (iii) “hunger” as susceptibility to internal or external hunger signs. Cronbach’s α coefficients [49] for cognitive restraint, disinhibition, and hunger were 0.805, 0.807, and 0.771, respectively.

All TFEQ items were scored either 0 or 1 point, leading to maximum sum scores of 21 points for cognitive restraint, 16 points for disinhibition, and 14 points for hunger. Higher scores indicate stronger characteristic values in the domains.

### Statistical analysis

Statistical analyses were performed using the R statistical software (v4.0.21; R Foundation for Statistical Computing, Vienna, Austria). In the whole sample, the reliability of the WSSQ-J (internal consistency) was estimated by calculating Cronbach’s α [49]. Confirmatory factor analysis (CFA) was then carried out with the lavaan package [50] to confirm that the factor structure of the WSSQ-J was the same as that of the subscales of the original WSSQ. Subsequently, the criterion-related validity of the WSSQ-J was examined through correlation analyses between scores on the WSSQ-J and the TFEQ-J. Since the Shapiro–Wilk test showed that the total and subscale scores of the WSSQ-J and the TFEQ-J were not normally distributed (*p* < 0.05), the Kendall’s rank correlation test was used. Finally, the association between the WSSQ-J and demographic characteristics was examined. First, sex differences in the WSSQ-J scores were examined by comparing the total and subscale scores. Since the Shapiro–Wilk test showed that the total and subscale scores of the WSSQ-J were not normally distributed in males and females (*p* < 0.05), the Wilcoxon rank sum test was used. In addition, correlations between the WSSQ-J and BMI or age were also tested. Given that BMI had two missing values, these data were omitted from this correlation analysis.

Since the original WSSQ was designed to assess WBI for people who are overweight or obese [31], the reliability of the WSSQ-J was examined in the non-overweight or non-obese (BMI < 25) sample (n = 1256) to test whether measurement invariant of the WSSQ-J could be confirmed in the samples with this weight status. Internal consistency was estimated by calculating Cronbach’s α, and CFA was performed to confirm that the WSSQ-J would have a two-factor structure similar to the original WSSQ.

## Results

### Characteristics of the WSSQ-J

The Kendall’s rank correlation showed that both the fear of enacted stigma (τ (correlation coefficient) = 0.730, *p* < 0.001) and self-devaluation (τ = 0.826,* p* < 0.001) subscales were positively correlated with the total scale score. In addition, these two subscales were positively correlated with each other (τ = 0.526, *p* < 0.001).

### Internal consistency

Cronbach’s α coefficients for the total WSSQ-J scale and the fear of enacted stigma and self-devaluation subscales were 0.917, 0.818, and 0.915, respectively. Cronbach’s alpha of 0.70 and above is acceptable [51]. Thus, the internal consistency of the WSSQ-J was determined to be good.

### Confirmatory factor analysis

**Fig. 1:**
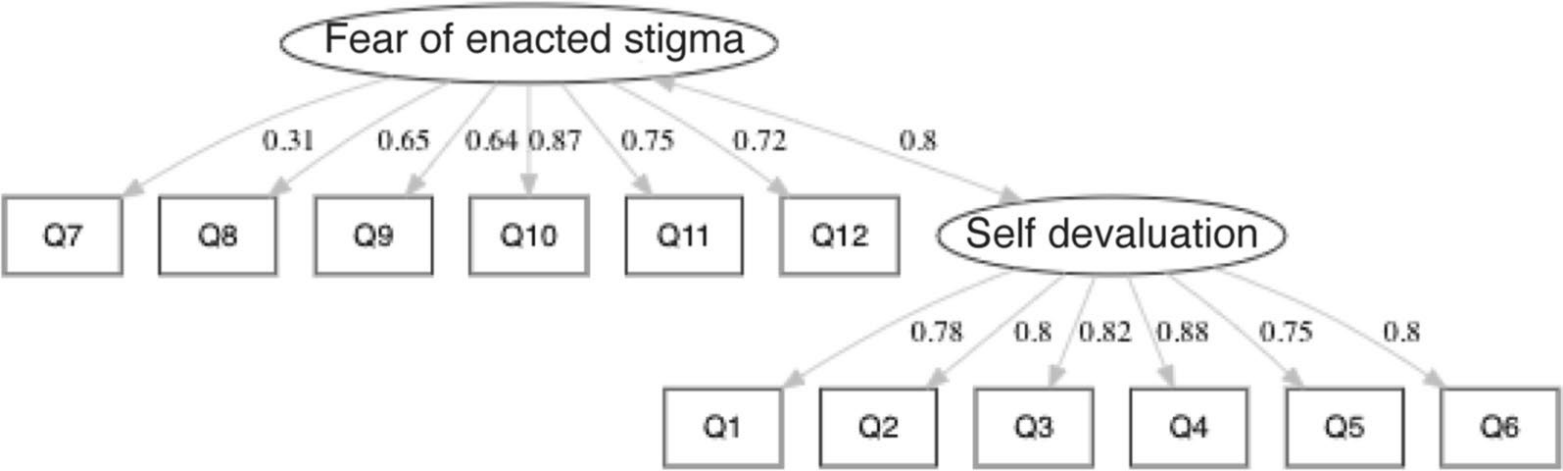
Results from confirmatory factor analyses of the WSSQ-J. The double-headed arrow indicates the standardized model covariance and other arrows indicate standardized factor loadings

The CFA assessed the model fit of the two-factor model [*χ*^2^ = 604.81 (*df* = 53, *p* < 0.001)]. The comparative fit index (CFI) was 0.945 and the Tucker-Lewis index (TLI) was 0.932. Cut-off values for the CFI and TLI are > 0.95 [52]. Therefore, these are approximately good fit indices for internal validity. The root mean square error of approximation (RMSEA) was 0.085 (90% confidence interval (CI) [0.079, 0.091]) and the standardized root mean square residual (SRMR) was 0.040. An RMSEA of between 0.08 to 0.10 indicates a mediocre fit and below 0.06 shows a good fit, and an SRMR of less than 0.05 indicates a well-fitting index [52]. Thus, these values suggest that the two-factor model showed generally satisfactory goodness-of-fit. Results of the CFA of the WSSQ-J are presented in Fig. 1.

The standardized factor loadings were 0.31–0.87 for the fear of enacted stigma, and 0.75–0.88 for the self-devaluation. As item 7 did not load well onto the fear of enacted stigma (factor loading = 0.31), this item was removed; we performed the CFA to examine whether the model fit would improve. Removing this item did not significantly improve the model fit (Supplemental Results).

### The criterion-related validity of the WSSQ-J

The fear of enacted stigma, self-devaluation subscales, and total WSSQ-J scores showed significant positive correlations with scores for the cognitive restraint of eating, disinhibition, and hunger subscales of the TFEQ-J (*p*_*Bonferroni-corrected*_ < 0.05), although the fear of enacted stigma showed a positive correlation with the cognitive restraint of eating at a trend level (*p*_*Bonferroni-corrected*_ = 0.054) (Table 2).

**Table 2:** Bivariate associations

Measure	WSSQ-J (total)	WSSQ-J (self-devaluation)	WSSQ-J (fear of enacted)
WSSQ-J^a^ (total)	1	0.826**	0.730**
WSSQ-J^a^ (self-devaluation)	0.826**	1	0.526**
WSSQ-J^a^ (fear of enacted)	0.730**	0.526**	1
Age^a^	0.016	0.033	− 0.012
BMI^a^	0.307**	0.339**	0.205**
Sex^b^	203,196**	197,828**	215,944*
TFEQ-J^a^ (cognitive restraint)	0.051*	0.052*	0.043
TFEQ-J^a^ (disinhibition)	0.402**	0.419**	0.302**
TFEQ-J^a^ (hunger)	0.262**	0.260**	0.217**

*TFEQ-J* Japanese version of the three-factor eating questionnaire, *WSSQ-J* Japanese version of the weight self-stigma questionnaire

*p < 0.05; **p < 0.01

^a^τ value of the Kendall’s rank correlation test

^b^W value of the Wilcoxon rank sum test

### Associations between the WSSQ-J and demographics

The total and subscale scores of the WSSQ-J in females were significantly greater than those in males (*ps* < 0.004). The Kendall’s rank correlation test showed that the fear of enacted stigma, self-devaluation subscales, and total WSSQ-J scores were positively correlated with BMI (*p* < 0.001), but not with age (*ps* > 0.070) (Table 2).

### Measurement invariant of the WSSQ-J in the non-overweight or non-obese sample

In the non-overweight or non-obese sample, Cronbach’s α coefficients for the total WSSQ-J scale and the fear of enacted stigma and self-devaluation subscales were 0.912, 0.806, and 0.914, respectively. Overall, the internal consistency of the WSSQ-J was determined to be good [51].

In the non-overweight or non-obese sample, the CFA assessed the model fit of the two-factor model [χ^2^ = 525.40 (df = 53, p < 0.001)]. The CFI was 0.944 and the TLI was 0.930. These are good fit indices for internal validity [52]. The RMSEA was 0.084 (90% CI [0.078, 0.091]) and the SRMR was 0.042. These values suggest that the two-factor model showed satisfactory goodness-of-fit [52].

## Discussion

To the best of our knowledge, this study is the first to validate and report on the WSSQ-J. A total of 1454 Japanese participants were included to validate the WSSQ-J. Cronbach’s α for the WSSQ-J total and the two subscales showed that the internal reliability of the WSSQ-J was satisfactory for research use [51]. The CFA showed that the two-factor model showed generally satisfactory goodness-of-fit, which was consistent with previous research [31]. Furthermore, in line with the previous report about the original version of the WSSQ [31], the total and subscales of the WSSQ-J showed positive correlations with the subscales of the TFEQ-J. In addition, the total and subscale scores of the WSSQ-J in females were greater than those in males, indicating that females experience higher levels of weight-related stigma. Moreover, BMI was positively correlated with the subscale and total scores of the WSSQ-J. In the non-overweight or non-obese sample, the reliability of the WSSQ-J was satisfactory; the CFA showed that the WSSQ-J had a two-factor structure similar to the original WSSQ [31], although the original WSSQ was designed for people who are overweight or obese. Overall, the results indicate that the WSSQ-J has good reliability and criterion-related validity, and can be a useful tool to measure weight-related self-stigma in Japanese adults.

Item 7 did not load well onto the fear of enacted stigma in this study. In the previous research about the original WSSQ, the factor loading of item 7 was 0.67 [31]. In the young adult samples with a broad range of weight status from underweight to overweight, the factor loading of item 7 was 0.59 [35] and 0.58 [36]. Compared to these previous studies, the factor loading of item 7 was low in the current study (0.31). Item 7 states, “I feel insecure about others’ opinions of me.” This item is practically irrelevant to individuals’ weight status, unlike other items constituting the fear of enacted stigma subscale (Items 8–12). Although such subscale is supposed to contain items that pertained to fear of enacted stigma and discrimination related to weight, item 7 does not necessarily involve discrimination related to weight. Therefore, item 7 did not load well onto the fear of enacted stigma in the current study.

Consistent with the original report of the WSSQ [31], greater BMI was correlated with higher WSSQ-J subscale and total scores. However, unlike the original WSSQ report with overweight or obese samples [31], in this study, fear of enacted stigma has a lower correlation coefficient with BMI compared to self-devaluation. This is consistent with a previous study with non-overweight samples, which showed that fear of enacted stigma had a lower correlation coefficient with BMI compared with the correlation coefficient between self-devaluation and BMI [35]. Individuals with greater BMI would perceive greater weight stigma [12, 21], and experiencing more weight discrimination from others likely leads to more internalization in people with greater BMI [15]. Thus, the perceived weight stigma in the non-overweight samples could differ from that in overweight or obese samples. Therefore, the association between fear of enacted stigma and BMI in non-overweight samples could also differ from that in overweight or obese samples.

People with greater BMI show higher discrepancies between their current and desired weight [53], and such increased discrepancies would lead to a higher desire to lose weight. However, WBI is connected to disordered eating, such as emotional, uncontrolled, and binge eating [19–22]. Moreover, individuals with higher WBI are more likely to report coping with experiences of weight stigma by eating [16]. In fact, in the current study, greater WSSQ-J scores were positively associated with the TFEQ-J disinhibition subscale, which indicates loss of control in food intake, and the TFEQ-J hunger subscale, which indicates susceptibility to internal or external hunger signs. These TFEQ-J subscales have been shown to be associated with maladaptive eating patterns [54–56]. Thus, in the current study, some people with greater WBI would have disordered eating. Therefore, ironically, when people with greater WBI seek weight loss, they may experience more disordered eating and have greater difficulty losing weight compared to people with less WBI.

In line with some previous reports [15, 16], the WSSQ-J subscale and total scores in females were greater than those in males. Therefore, females are more likely to internalize weight stigma [15, 16]. In Japan, women are still expected to play a supportive and modest role to confirm men’s higher status over women in the vertical societal hierarchy [38]. In fact, a study showed that, compared to women in western countries, Japanese women spoke with higher pitch, which reflects physical and psychological powerlessness (short, weak, dependent, and modest), to express sociocultural expectations of femininity [57]. Because of these socioculturally standardized gender roles, Japanese women are likely to have lower self-esteem and self-assertion, and have high regard for slimness as a norm of physical beauty [38]. These characteristics would be associated with WBI [58, 59] and could lead to the onset of eating disorder symptoms [38]. The prevalence of eating disorders is higher in females compared to males in Japan [37, 38]. Given that WBI is connected with disordered eating [15, 16, 19, 21, 22], which would be related to eating disorders [41, 42], and sociocultural pressures regarding appearance [43], the effects of gender on the role of WBI in eating disorders could be different between western countries and Japan. Future research should include more diverse samples to examine sociocultural effects on associations between gender and WBI.

Susceptibility to weight bias would be different between people in western countries and Japan. Although Japanese culture has become more individualistic over time, traditional collectivism persists in Japan [60]. A previous meta-analytic study showed that collectivism significantly moderated the process of stigma internalization [61]. Compared with western countries, Japanese people strongly dislike people who are overweight or obese over lean people; thus, weight bias toward individuals with greater BMI could be stronger in Japan [62]. Overall, Japanese people would also tend to internalize weight bias. Hence, it would be favorable to examine differences in the effect of the attitudes towards being overweight and obesity on weight bias internalization across countries and cultures.

### Strength and limits

This study has limitations that must be considered in future studies. First, the test–retest reliability of the WSSQ-J was not examined and should be tested in future research. Second, the current study did not include adolescents or older adults. Although there was no significant association between WSSQ-J score and age in the current sample, previous research indicates that younger people would have higher WBI [15]. Future studies should include a wide range of age groups to test the effect of WBI. Third, self-reported weight and height were used to calculate BMI. Given that self-reported height tends to be overestimated while self-reported weight tends to be underestimated [63], BMI calculated from self-reported data is likely to be lower than the measured data [63, 64]. However, since large cohort studies suggested that BMI computed from self-reported weight and height was an adequately valid measure in large epidemiological studies [63, 64], the reliability of the current results is assumed. Fourth, although the WSSQ-J showed a good reliability and two-factor structure in this study, measurement invariant of the WSSQ-J was not tested in individuals who are overweight and obese. The current sample included only 196 participants who are overweight or obese, and this sample size is insufficient for CFA [65, 66]. Since the original WSSQ was constructed for people who are overweight or obese [31], the constructs of the WSSQ-J would be different between overweight and non-overweight samples; moreover, measurement invariant of the WSSQ-J should be tested in individuals who are overweight or obese in future studies.

A strength of the present study is that, to the best of our knowledge, this is the first study that has examined psychometric properties of a Japanese version of the WSSQ. In addition, this study was conducted with a sufficient sample size compared with previous similar studies [34–36]. The present study also used a rigorous translation procedure to ensure the linguistic validity of the WSSQ-J.

## What is already known on this subject?

The WSSQ is widely used to assess WBI and has the two-factor structure and extensive psychometric validation. Psychometric properties and a two-factor structure of the WSSQ were confirmed in English-, Arabic-, Chinese-, French-, German-, Italian-, Persian-, and Turkish-speaking samples.

## What your study adds?

By partially replicating the original study of the WSSQ, this study has confirmed that the WSSQ-J has good reliability and criterion-related validity. Therefore, the WSSQ-J can be a reliable and useful tool for assessing WBI among the Japanese population.

## Supplementary Information

Below is the link to the electronic supplementary material.

Supplementary file 1 (DOCX 17 KB)

## Data Availability

The anonymized data presented in this study are available upon request. Please contact the corresponding author (Y.N.).
