# Supplementary material for: Developing and validating a Japanese version of the Weight Self-Stigma Questionnaire
Source: Eat Weight Disord. 2023 May 17;28(1):44. doi: 10.1007/s40519-023-01573-0 (PMC10191951; doi:10.1007/s40519-023-01573-0)
Supplement: Supplementary file 1 — (DOCX 17 KB) [file 40519_2023_1573_MOESM1_ESM.docx]

**Supplemental Results**

**Confirmatory factor analysis without item 7**

The CFA assessed the model fit of the two-factor model [χ^2^ = 560.42 (df = 43, p < 0.001)]. The comparative fit index (CFI) was 0.948 and the Tucker-Lewis index (TLI) was 0.934. The root mean square error of approximation (RMSEA) was 0.091 (90% confidence interval (CI) [0.084, 0.098]) and the standardized root mean square residual (SRMR) was 0.041. Thus, the two-factor model still showed generally satisfactory goodness-of-fit after removing the item 7, and removing item 7 did not improve model fit significantly.

**Table S1** English and Japanese back-translated items from the WSSQ

| Items | Subscales | English items | Japanese items |
| --- | --- | --- | --- |
| 1 | Self-devaluation | I’ll always go back to being overweight | 私はいつも肥満に戻ってしまう |
| 2 | Self-devaluation | I caused my weight problems | 私のせいで自分の体重の問題が起こっている |
| 3 | Self-devaluation | I feel guilty because of my weight problems | 自分の体重の問題に罪の意識を感じる |
| 4 | Self-devaluation | I became overweight because I’m a weak person | 私は弱い人間だから肥満になってしまった |
| 5 | Self-devaluation | I would never have any problems with weight if I were stronger | もし、自分がもっと強い人間だったら、体重の問題に悩まされることはなかっただろう |
| 6 | Self-devaluation | I don’t have enough self-control to maintain a healthy weight | 私には健康的な体重を維持するための自制心が足りない |
| 7 | Fear of enacted stigma | I feel insecure about others’ opinions of me | 自分に対する他人の意見に自信がない |
| 8 | Fear of enacted stigma | People discriminate against me because I’ve had weight problems | 私は体重の問題を抱えているので、みんなは私のことを差別する |
| 9 | Fear of enacted stigma | It’s difficult for people who haven’t had weight problems to relate to me | 体重に悩まされたことのない人は私に共感を持ちにくい |
| 10 | Fear of enacted stigma | Others will think I lack self-control because of my weight problems | 私は体重の問題を抱えているので、他の人は私の自制心が足りないと思うだろう |
| 11 | Fear of enacted stigma | People think that I am to blame for my weight problems | みんなは、私の体重の問題は私に責任があると思っている |
| 12 | Fear of enacted stigma | Others are ashamed to be around me because of my weight | 私の体重の問題のせいで、私の周りの人は私と一緒にいることを恥じている |
